# Degradation and inactivation of Shiga toxins by nitrogen gas plasma

**DOI:** 10.1186/s13568-017-0380-7

**Published:** 2017-04-07

**Authors:** Akikazu Sakudo, Yuichiro Imanishi

**Affiliations:** 1 Laboratory of Biometabolic Chemistry, School of Health Sciences, Faculty of Medicine, University of the Ryukyus, 207 Uehara, Nishihara, Okinawa 903-0215 Japan; 2 NGK Insulators Ltd., 2-56 Suda-cho, Mizuho, Nagoya, 467-8530 Japan

**Keywords:** Non-thermal gas plasma, Degradation, Discharge, Static induction thyristor, Verotoxin, Shiga toxin

## Abstract

Shiga toxin (Stx)-producing *Escherichia coli* (STEC) leads to food poisoning by causing hemorrhagic colitis and hemolytic uremic syndrome. Some STEC produce Shiga toxin 1 (Stx1) and/or Shiga toxin 2 (Stx2), a relatively stable protein toxin, necessitating the development of an efficient inactivation method. Here we applied a nitrogen gas plasma apparatus to the inactivation of Stx. Samples of Stx1 and Stx2 were treated with a nitrogen gas plasma generated by a plasma device using a short high-voltage pulse applied by a static induction thyristor power supply at 1.5 kpps (kilo pulse per second). The recovered Stx samples were then analyzed for immunological and biological activities. Immunochromatography demonstrated that Stx1 and Stx2 were degraded by the gas plasma. Quantification by enzyme-linked immunosorbent assay (ELISA) showed that both toxins were efficiently degraded to less than 1/10th of their original concentration within 5 min of treatment. Western blotting further showed the gas plasma treatment degraded the A subunit, which mediates the toxicity of Stx. Moreover, an assay using HEp-2 cells as an index of cytotoxicity showed that gas plasma treatment reduced the toxic activity of Stx. Therefore, nitrogen gas plasma might be an efficient method for the inactivation of Stx.

## Introduction

The virulence of Shiga toxin (Stx)-producing *Escherichia coli* (STEC), such as *E. coli* O157:H7 and other serotypes, including their most dangerous subset, enterohemorrhagic *E. coli* (EHEC), depends on the production of Stx (Gyles 2007; Hunt 2010). The toxins inhibit protein synthesis and are therefore deleterious to humans, leading to life-threating complications including hemorrhagic colitis (HC) and hemolytic uremic syndrome (HUS) (Boerlin et al. 1999; Scallan et al. 2011). These illnesses are known as food-borne diseases, resulting from the ingestion of food contaminated with STEC and their toxins. To improve the quality and safety of the food supply, it is necessary to minimize toxic compounds in foods. Thus, technologies to degrade and/or inactivate Stx are needed.

Stx produced by STEC comprise two major groups; namely, Shiga toxin 1 (Stx1) and Shiga toxin 2 (Stx2). Stx1 has identical characteristics to Stx produced by *Shigella dysenteriae* in terms of immunological, physical, chemical, and biological properties (Takao et al. 1988). Stx2 has 55% nucleotide sequence identity to Stx1 but differs substantially in its physiological properties (Jackson et al. 1987; Scotland et al. 1985). Stx1 and Stx2 each comprise an A subunit monomer, which plays a role in toxicity, and a pentamer of B subunits, which are involved in binding to a cellular receptor, globotriaosylceramide (Gb3) (Lingwood et al. 1987; Stein et al. 1992; Fraser et al. 1994). Stx2 is known to be highly virulent, and is several orders of magnitude more toxic than Stx1 (Nataro and Kaper 1998; Pickering et al. 1994; Manning et al. 2008). Furthermore, Stx2 is relatively heat stable and is not inactivated by pasteurization (Rasooly and Do 2010).

Several compounds have been reported to inhibit Stx1 and Stx2 (Sugita-Konishi et al. 1999; Kulkarni et al. 2010; Rasooly et al. 2010; Quinones et al. 2009; Friedman et al. 2013). However, it remains unclear whether these anti-Stx1 and anti-Stx2 compounds cause irreversible inactivation or whether they have indirect effects, such as inhibiting toxin release or affecting the digestive tract of the host (Friedman and Rasooly 2013).

Recently, the effectiveness of gas plasma technology in eradicating various types of microbes such as viruses, bacteria and fungi has been demonstrated (Fridman 2012; Laroussi et al. 2012; Shintani et al. 2010). In this regard, gas plasma technology is a promising method for achieving disinfection and sterilization in a broad range of applications. In particular, gas plasma technology provides an excellent platform for food control, preventing the spread of harmful microbes. For example, Schlüter’s group has demonstrated inactivation of Stx-producing *E. coli* using an atmospheric pressure plasma jet (Baier et al. 2015).

The gas plasma can be artificially generated by various types of discharge, including arc, corona, direct current, glow, high/low frequency, micro, pulse, and streamer. Further developments in gas plasma technology are likely to enable broader applications including the detoxification of toxins. Although Park’s group and others have worked on the inactivation of lipopolysaccharide using a different technology for producing gas plasma (Park et al. 2007; Shintani et al. 2007), there are no reports showing the inactivation of Stx using gas plasma.

Recently, we successfully generated nitrogen gas plasma by applying a short-time high-voltage pulse to nitrogen gas as an inert gas, using a static induction (SI) thyristor power supply (Sakudo et al. 2013a). Bacterial spores, vegetative bacteria, and viruses are all inactivated by the nitrogen gas plasma (Sakudo et al. 2014; Maeda et al. 2015). Several factors contribute to the thermal and non-thermal mechanism of inactivation, such as heat, ultraviolet (UV) radiation, and reactive chemical species generated during operation of the nitrogen gas plasma device. Among them, reactive chemical species may be the most important factor for inactivation of bacteria and viruses (Maeda et al. 2015; Sakudo et al. 2017).

Here, based on these previous observations, we have extended our research to examine the potential use of nitrogen gas plasma for the inactivation of Stx. The effect of nitrogen gas plasma treatment on Stx was investigated by biochemical analyses and a bioassay using cell culture. The potential mechanisms of Stx inactivation by the gas plasma are then discussed.

## Materials and methods

### Exposure of Stx samples to gas plasma

Stx1 from *E. coli* O157:H7 and Stx2 from *E. coli* O157:H7, which were solubilized at 0.1 mg/ml in 0.1 M Tris–HCl buffer (pH 8.6), 0.1 M NaCl, 0.001% polyvinyl alcohol after purification by affinity chromatography using Gb3 and 0.45 μm filtration, were purchased from Nakalai Tesque (Kyoto, Japan). The Stx1 and Stx2 solutions were each diluted with phosphate-buffered saline (PBS) to 1 μg/ml, and a 20 μl aliquot was dropped onto a coverslip (24 mm × 60 mm Thickness No. 1, 0.12–0.17 mm, Matsunami Glass Industries, Ltd., Osaka, Japan) and air-dried. The nitrogen gas plasma was produced by a BLP-TES (bi-polar and low-pressure plasma-triple effects sterilization) device (NGK Insulators, Ltd.) using a short-time high-voltage pulse generated by an SI thyristor, which was used as a pulsed power supply. The coverslip containing the Stx samples was placed in the device’s chamber on a grid of electrodes, comprising a cathode electrode (earth electrode) between two anode electrodes (high voltage electrodes). The chamber box was then decompressed and degassed, and nitrogen gas (99.9995%, Okano, Okinawa, Japan) was introduced at a flow rate of approximately 10 L/min. The pressure in the box was maintained at about 0.5 atmospheres, and the plasma was discharged at 0–1.5 kpps (kilo pulse per second). Nitrogen gas plasma-treated and control untreated samples were recovered from the coverslip by reconstitution in 20 μl of PBS. The recovered solutions were subjected to biochemical and immunological analysis.

### Immunochromatography

Stx1 and Stx2 in the recovered samples were detected by immunochromatography using the NH Immunochromato VT1/2 kit (NH Foods Ltd., Osaka, Japan) in accordance with the manufacturer’s instructions. The kit produces two lines: development of a control line indicates that the strip has functioned properly, whereas development of a test line indicates the presence of Stx1 or Stx2. The kit can detect Stx1 at 2.5 ng/ml, and Stx2 at 1.25 ng/ml (Yonekita et al. 2012).

### Enzyme-linked immunosorbent assay (ELISA)

Stx1 and Stx2 in the recovered samples were quantified by using an ELISA kit, RIDASCREEN^®^ Verotoxin (R-Biopharm AG, Darmstadt, Germany) in accordance with the manufacturer’s instructions. The concentration of Stx1 and Stx2 was quantified by measuring absorbance at 450 nm relative to respective Stx1 and Stx2 standards.

### Calculation of the detection limit

The limit of detection in the ELISA was calculated according to Miller’s study (Miller and Miller 2010). Equation 1 defines the detection limit (*y*), which indicates whether a sample contains a certain Stx based on the average (*y*_B_) and standard deviation (S_B_) of the signal from a blank control (signal of samples without Stx). The calculated intercept is used as an estimate of *y*_B_. Equation 2 is used to estimate S_B_, or statistics S_*y*/*x*_, which estimates the random error in the *y* direction (Miller and Miller 2010). The *ŷ*_i_ values are the points corresponding to the individual *x* values on the calculated regression line:

1 $$y = y_{\text{B}} + 3{\text{S}}_{\text{B}}$$

2 $${\text{S}}_{y/x} = \left\{ {\frac{{\sum\nolimits_{\text{i}} {({\text{y}}_{\text{i}} - {\hat{\text{y}}}_{\text{i}} )^{2} } }}{{{\text{n}} - 2}}} \right\}^{1/2}$$

### Western blotting

Each recovered sample was added to an equal volume of 2× sodium dodecyl sulfate (SDS) gel-loading buffer [90 mM Tris–HCl (pH 6.8), 10% mercaptoethanol, 2% SDS, 0.02% bromophenol blue, and 20% glycerol] and boiled for 5 min. The proteins were then resolved by SDS–polyacrylamide gel electrophoresis (PAGE) before being electroblotted onto a polyvinylidene difluoride (PVDF) membrane (Hybond-P; Amersham-Pharmacia Biotech, Piscataway, NJ, USA) for 60 min at 15 V. Blots were treated with 5% skimmed milk for 1 h at room temperature and then incubated with a rabbit polyclonal anti-Stx1 antibody (Cat No. 64-025, BioAcademia, Osaka, Japan) or a mouse monoclonal anti-Stx2 antibody (Cat No. 20273-04, Nakalai Tesque) in PBS containing 0.1% Tween 20 (PBS-T) and 0.5% skimmed milk for 1 h at room temperature. After three washes with PBS-T, the membrane was incubated in horseradish peroxidase (HRP)-conjugated anti-rabbit IgG or anti-mouse IgG (Jackson ImmunoResearch Laboratories, Inc., West Grove, PA, USA) in PBS-T and 0.5% skimmed milk for 1 h at room temperature. After three washes with PBS-T, the proteins were detected by using an enhanced chemiluminescence detection kit (Amersham-Pharmacia Biotech) and an Ez-Capture MG imaging system (ATTO Corp., Tokyo, Japan).

### Cell assay

The activity of Stx1 and Stx2 was measured by previously described methods (Jones et al. 2000) with slight modification. In brief, the index of cytotoxic activity of Stx1 and Stx2 was measured in HEp-2 cells, cultured in Minimum Essential Medium (MEM) containing 10% fetal calf serum (FCS). The recovered Stx samples were 50-fold diluted with serum-free MEM and then added to 1 × 10^4^ HEp-2 cells at 100 μl/well in a 96-well microtiter plate. After incubation at 37 °C for 24 h for Stx1 and for 48 h for Stx2, cell viability was measured by using the Cell Counting kit-8 (Dojindo, Kumamoto, Japan). In accordance with the manufacturer’s instructions, 10 μl of cell counting kit-8 solution was added to the microplate, which was then incubated at 37 °C in 5% CO_2_. Absorbance was determined at 450 nm relative to a reference wavelength of 630 nm using a microplate reader (Model 680; Bio-Rad, Hercules, CA, USA).

### Statistical analysis

Results are reported as the mean ± standard deviation. Statistical analysis of differences was performed by non-repeated measures analysis of variance (ANOVA), followed by the Bonferroni correction.

## Results

To investigate the effect of nitrogen gas plasma on Stx, 20 μl of a 1 μg/ml solution of Stx1 or Stx2 was spotted onto a coverslip, which was placed on the earth electrode of the BLP-TES device, and subjected to nitrogen gas plasma treatment (1.5 kpps, 0, 5, 15, and 30 min). Stx1 and Stx2 were then recovered from the treated spots, and subjected to various analyses.

**Fig. 1:**
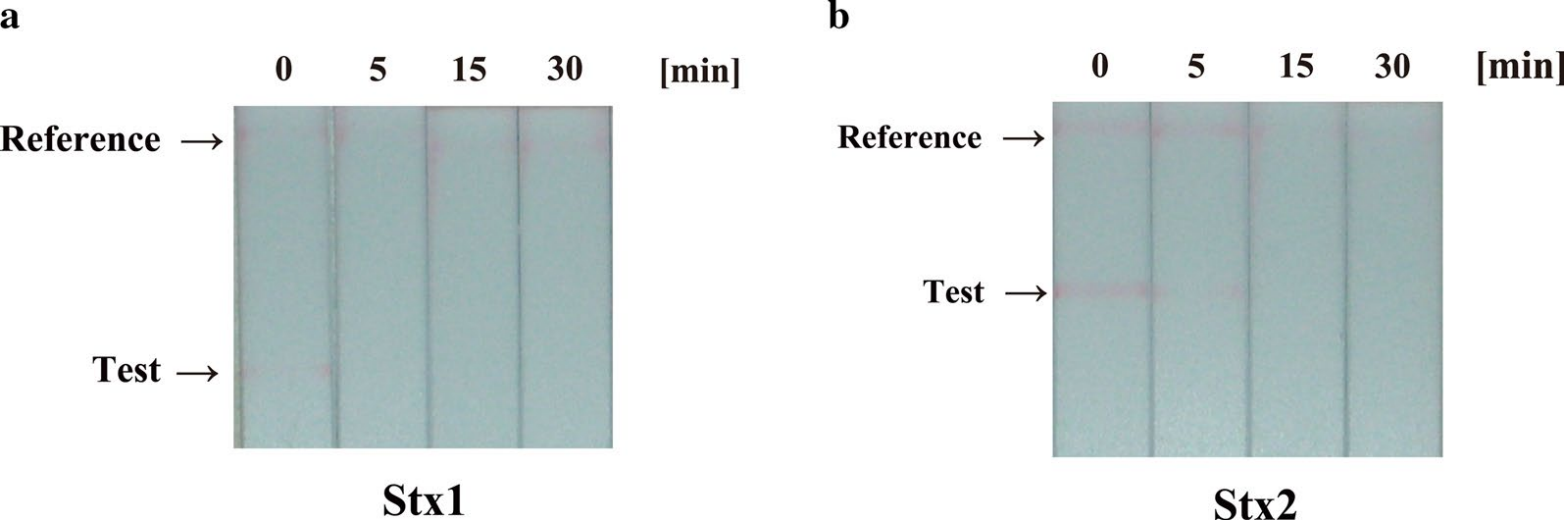
Immunochromatography of Shiga toxin 1 (Stx1) and Shiga toxin 2 (Stx2) after nitrogen gas plasma treatment. A coverslip containing dried spots from 20 μl aliquots of a 1 μg/ml solution of Shiga toxins (Stx) including Stx1 (**a**) and Stx2 (**b**) was treated with nitrogen gas plasma using a bi-polar and low-pressure plasma-triple effects sterilization (BLP-TES) device (1.5 kpps) for 0, 5, 15, or 30 min. The recovered Stx1 and Stx2 samples were analyzed by immunochromatography using an NH immunochromato VT1/2 kit (NH Foods Ltd., Osaka, Japan). Test lines for Stx1 and Stx2, and* reference lines* for the internal control are indicated by *arrows*

First, the treated Stx1 and Stx2 were subjected to immunochromatography (Fig. 1). The results showed that the test lines of Stx1 were diminished by gas plasma treatment for 5, 15 and 30 min, as compared with the untreated sample (0 min) (Fig. 1a). In the case of Stx2, test lines were detected in the untreated sample (0 min) and the sample treated for 5 min, but were diminished in samples treated for 15 and 30 min (Fig. 1b).

**Fig. 2:**
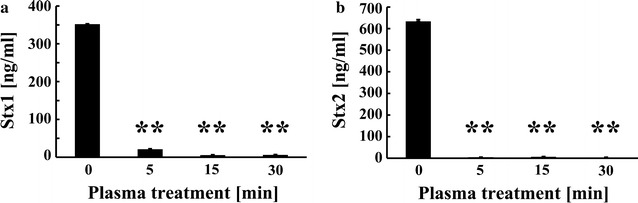
Quantitative measurement of Stx1 and Stx2 by an enzyme-linked immunosorbent assay (ELISA) after nitrogen gas plasma treatment. A coverslip containing dried spots from 20 μl aliquots of a 1 μg/ml solution of Stx1 (**a**) and Stx2 (**b**) was treated with nitrogen gas plasma using BLP-TES device (1.5 kpps) for 0, 5, 15, and 30 min. The recovered samples were subjected to ELISA using RIDASCREEN^®^ Verotoxin (R-Biopharm AG, Darmstadt, Germany) to quantify Stx1 and Stx2. Values were considered significantly different from the untreated control (0 min) when verified by non-repeated measures ANOVA, followed by the Bonferroni correction (**p < 0.01)

Next, the gas plasma-treated Stx1 and Stx2 samples were subjected to ELISA (Fig. 2), which provides a quantitative and more sensitive and accurate analysis than immunochromatography. The detection limit of the ELISA was calculated to be 0.3279 ng/ml for Stx1 and 0.6540 ng/ml for Stx2. The results of the ELISA (mean ± standard deviation) showed that the treatment of Stx1 and Stx2 with nitrogen gas plasma at 1.5 kpps decreased the toxin levels by more than 90%: Stx1 decreased from 351.98 ± 19.20 ng/ml at 0 min of treatment to 21.24 ± 0.65 ng/ml at 5 min of treatment, and Stx2 decreased from 633.56 ± 16.33 ng/ml at 0 min to 4.27 ± 0.91 ng/ml at 5 min. Plasma gas treatment for 15 min led to 5.60 ± 1.14 ng/ml of Stx1 and 6.18 ± 3.38 ng/ml of Stx2, whereas that for 30 min led to 6.03 ± 1.84 ng/ml of Stx1 and 3.71 ± 1.13 ng/ml of Stx2. Thus, the concentration of Stx1 and Stx2 at 5, 15, and 30 min was almost at the detection limit of the ELISA assay.

**Fig. 3:**
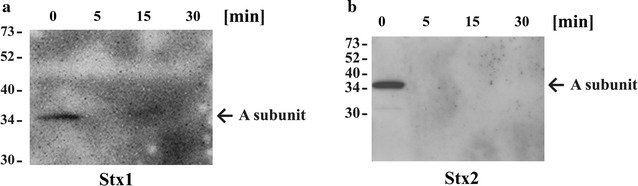
Western blotting of Stx1 and Stx2 after nitrogen gas plasma treatment. A coverslip containing dried spots from 20 μl aliquots of a 1 μg/ml solution of Stx1 (**a**) and Stx2 (**b**) was treated with nitrogen gas plasma using BLP-TES device (1.5 kpps) for 0, 5, 15, or 30 min. The recovered samples were subjected to Western blotting using antibody recognizing the A subunit of Stx

Western blotting was also performed to investigate the effect of nitrogen gas plasma on Stx1 and Stx2 (Fig. 3). This analysis showed that the band corresponding to the A subunit of Stx1 (approximately 35 kDa) and Stx2 (approximately 35 kDa) (Russo et al. 2014) diminished after nitrogen gas plasma treatment for 5, 15, and 30 min, as compared with that of the untreated sample (0 min).

**Fig. 4:**
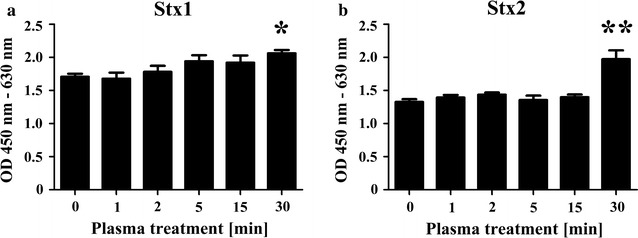
Decreasing cell toxicity of Stx1 and Stx2 after nitrogen gas plasma treatment. A coverslip containing dried spots from 20 μl aliquots of a 1 μg/ml solution of Stx1 (**a**) and Stx2 (**b**) was treated with nitrogen gas plasma using BLP-TES device (1.5 kpps) for 0, 1, 2, 5, 15, and 30 min. The recovered samples were diluted 50-fold with minimum essential medium (MEM) and then added to HEp-2 cells at 100 μl/well in a microtiter plate. After incubation at 37 °C for 24 h for Stx1 and for 48 h for Stx2, the viability of HEp-2 cells was monitored using a Cell counting kit 8 (Dojindo, Kumamoto, Japan). The activity of Stx, which was measured on the basis of cell toxicity of Stx to HEp-2 cells, was significantly inhibited, as compared with no treatment (0 min), by 30 min of nitrogen gas plasma treatment using BLP-TES (*p < 0.05, **p < 0.01)

The observation of Stx1 degradation by gas plasma treatment was further supported by a cell assay (Fig. 4). Because Stx1 and Stx2 have cytotoxic activity against HEp-2 cells, the change in cell viability after incubation with the treated samples was used as an index of the activity of Stx1 and Stx2. The viability of cells incubated with untreated Stx1 (0 min in Fig. 4a) and Stx2 (0 min in Fig. 4b) was lower than that of cells incubated with gas plasma-treated samples, suggesting that the cytotoxic activity of Stx1 and Stx2 was reduced by increasing plasma treatment. Notably, cells incubated with samples subjected to gas plasma treatment for 30 min showed significantly higher viability as compared with cells incubated with untreated samples (*p* < 0.05 for Stx1; *p* < 0.01 for Stx2). Therefore, nitrogen gas plasma treatment causes not only the degradation of Stx1 and Stx2, but also a reduction of their physiological activity.

## Discussion

The potential use of physical methods to degrade Stx can be exploited to improve food safety. Here, the potential application of nitrogen gas plasma to degrade Stx has been studied using a BLP-TES device. The maximum temperature in the chamber box of the BLP-TES device was 42 °C at 5 min, 70 °C at 15 min, and 75 °C at 30 min (Sakudo et al. 2013b). Our study shows that during inactivation of Stx1 and Stx2 by the BLP-TES device, the sample temperature did not increase above 80 °C even after prolonged exposure for 30 min. Burk et al. (2003) previously showed that heat treatment above 80 °C for 10 min can be used to inactivate Stx. A more recent study reported only a slight decrease in activity of Stx2 after heat treatment at 60 °C for 1 h, although a significant reduction in activity was observed after treatment at 80 °C for 1 h (He et al. 2012). As the effective temperature for inactivation is close that seen during gas plasma treatment using the BLP-TES device, detailed quantitative and comparative studies are required to ascertain whether the inactivation mechanism of the gas plasma involves heat.

However, so far, the main mechanism of inactivation using the nitrogen gas plasma system of the BLP-TES device does not appear to involve exposure of the sample to elevated temperatures. A previous observation has suggested that the conformational changes in a protein induced by the nitrogen gas plasma are mediated by mechanisms distinct from those of heat degradation (Sakudo et al. 2013a). Because Stx1 and Stx2 are proteins, the conformational changes and degradation caused by the gas plasma may play important roles in their inactivation. In addition, the use of a more sensitive cell assay for Stx would be likely to detect the inactivating effect of the gas plasma on Stx at an earlier treatment time, because efficient degradation of Stx1 and Stx2 within 5 or 15 min of treatment was shown by all biochemical assays, including immunochromatography, ELISA, and Western blotting. Thus, further analysis using sensitive bioassays is required.

The Stx1 and Stx2 antibodies used in the Western blotting analysis recognize the A subunit of each toxin. The Western blotting data therefore suggest that the nitrogen gas plasma degrades the A subunit of Stx, which is known to elicit its toxic activity. Therefore, some factors generated during operation of the BLP-TES device such as the production of reactive chemical species, which have previously been shown to be the principal contributing reason for the inactivation of bacteria and viruses (Maeda et al. 2015; Sakudo et al. 2017), may be responsible for degrading Stx and their A subunits. Additional clarification of the mechanisms by which nitrogen gas plasma degrades Stx are required. Furthermore, in the present study, effective degradation of Stx was observed when a coverslip was used as the surface matrix during gas plasma treatment. Because the degradation efficiency and mechanisms may depend on the materials used for the surface matrix, further studies using other surface materials should be performed.

## Abbreviations

ANOVA: analysis of variance
BLP-TES: bi-polar and low-pressure plasma-triple effects sterilization
BRAIN: Bio-oriented Technology Research Advancement Institution
*E. coli*: *Escherichia coli*
EHEC: enterohemorrhagic *Escherichia coli*
ELISA: enzyme-linked immunosorbent assay
FCS: fetal calf serum
Gb3: globotriaosylceramide
HC: hemorrhagic colitis
HRP: horseradish peroxidase
HUS: hemolytic uremic syndrome
MEM: minimum essential medium
PAGE: polyacrylamide gel electrophoresis
PBS: phosphate-buffered saline
PBS-T: PBS containing 0.1% Tween 20
PVDF: polyvinylidene difluoride
SDS: sodium dodecyl sulfate
SI: static induction
STEC: Stx-producing *Escherichia coli*
Stx: Shiga toxin
Stx1: Shiga toxin 1
Stx2: Shiga toxin 2
UV: ultraviolet
